# Risk Factors for Poor Recovery After Total Knee Replacement Among Older Adults in the FORCE-TJR Cohort

**DOI:** 10.1093/geroni/igaa057.2714

**Published:** 2020-12-16

**Authors:** Thomas Laskow, Jiafeng Zhu, Brian Buta, Frederick Sieber, Karen Bandeen-Roche, Jeremy Walston, Patricia Franklin, Ravi Varadhan

**Affiliations:** 1 Johns Hopkins University, Baltimore, Maryland, United States; 2 School of Public Health, Johns Hopkins University, Baltimore, Maryland, United States; 3 Johns Hopkins Bloomberg School of Public Health, Baltimore, Maryland, United States; 4 Northwestern University Feinberg School of Medicine, Chicago, Illinois, United States

## Abstract

Total knee replacement (TKR) is a common procedure in older adults with broad variability in outcomes. We sought to identify factors that contribute to resilient outcomes in 7,239 older adults (age 60 or older) who underwent TKR in the TJR-FORCE, a prospective registry of total joint replacement. Outcomes utilized were bodily pain and physical component score (PCS) from the Short Form 36 Health Survey (SF-36), at pre-op, 1-year, and 2-year post-procedure. Participants were grouped according to their outcome trajectories as “improving”, “worsening”, “variable,” or “stable.” Multinomial regression (with 4 outcome categories) was used to evaluate demographic risk factors (age, gender, BMI, marital status, education, smoking history, comorbidity count, household income). Older age, larger comorbidity count, low-income, smoking, and being unmarried were significant risk factors for poor recovery (not “improving”) in terms of bodily pain and physical component score. Next steps include evaluating risk factors for resilience outcomes in prospective studies.

